# Epigenetic alterations in rheumatoid arthritis: multilayer mechanisms and translational opportunities

**DOI:** 10.3389/fimmu.2026.1871766

**Published:** 2026-09-01

**Authors:** Dominika Podgórska, Rafał Podgórski

**Affiliations:** 1 Department of Rheumatology, Faculty of Medicine, University of Rzeszów, Rzeszów, Poland; 2 Department of Medical Chemistry and Metabolomics, Faculty of Medicine, University of Rzeszów, Rzeszów, Poland

**Keywords:** biomarkers, chromatin accessibility, DNA methylation, epigenetics, histone modifications, non-coding RNA, rheumatoid arthritis

## Abstract

Rheumatoid arthritis (RA) is a chronic inflammatory disease driven by immune dysregulation, in which genetic susceptibility and environmental exposures promote persistent synovitis, progressive joint damage, and systemic comorbidities. Recent epigenomic studies show several recurring abnormalities. Many RA susceptibility variants lie outside protein-coding sequence and map to immune-cell and synovial fibroblast regulatory elements, linking inherited risk to enhancer activity, methylation quantitative trait effects, and distal gene control. Blood-based epigenome-wide association studies identify disease-associated DNA methylation signatures, but these signals require careful control for leukocyte composition, smoking, treatment exposure, and disease stage. RA fibroblast-like synoviocytes also display stable methylome remodeling, including relative hypomethylation at loci involved in inflammation, migration, matrix degradation, and apoptosis resistance, while TET3-associated 5-hydroxymethylcytosine has emerged as a functional contributor to chemokine production and invasive stromal behavior. Histone modifications, chromatin accessibility, and 3D genome organization define pathogenic regulatory states and connect non-coding risk loci to effector genes in immune and stromal compartments. Finally, miRNAs, lncRNAs, circRNAs, snoRNAs, extracellular RNAs, and m6A-related pathways add post-transcriptional and chromatin-linked layers with potential biomarker value. We synthesize these findings and discuss translational opportunities for diagnosis, stratification, flare monitoring, and therapeutic targeting, while emphasizing incomplete replication, uneven evidence across epigenetic layers, biospecimen variability, and the need for causal, longitudinal, cell-type-resolved validation.

## 1 Introduction

Rheumatoid arthritis (RA) is a systemic autoimmune disease characterized by chronic synovitis, progressive cartilage and bone destruction, and an increased risk of extra-articular manifestations. RA has long been recognized as clinically and biologically heterogeneous, with variation in autoantibody status, disease course, joint distribution, synovial histopathology, and therapeutic response (1). Despite major advances in biologic and targeted synthetic therapies, a substantial proportion of patients do not achieve sustained remission and many experience fluctuating disease activity over time (2, 3). Current models indicate that RA arises from interactions among heritable risk, environmental exposures, and tissue-context-dependent immune responses (4, 5). Human leukocyte antigen (HLA) class II variation, particularly HLA-DRB1 shared-epitope alleles, remains the strongest inherited component of seropositive RA, but many non-HLA loci also contribute to risk. Smoking and other exposures interact with these genetic backgrounds to promote autoantibody formation and establish a self-sustaining inflammatory circuit in the synovium (5, 6). This circuit is not maintained solely by leukocytes. Synovial fibroblasts integrate cytokine, T-cell, and B-cell signals and are now viewed as central tissue effectors and emerging therapeutic targets (7–11).

Genome-wide association studies (GWAS) have identified more than 100 RA susceptibility loci in large datasets. A major observation from these studies is that many associated variants do not change protein-coding sequence directly but instead lie in intronic, intergenic, or enhancer regions. Examples of non-HLA loci relevant to immune regulation include TNFAIP3, STAT4, TRAF1-C5, CD40, CCR6, IL2RA, IRF5, and IL6ST. These variants are therefore interpreted through regulatory mechanisms such as enhancer activity, expression and methylation quantitative trait loci and distal enhancer-promoter contacts, rather than through single-gene coding defects (6, 12, 13).

Epigenetic processes provide a mechanistic bridge between stable genetic risk and dynamic environmental cues. These processes include DNA methylation, histone modifications, chromatin accessibility, higher-order genome organization, regulatory non-coding RNAs, and RNA modifications such as N6-methyladenosine (m6A) (14–18). These layers are commonly studied using epigenome-wide methylation arrays; bisulfite sequencing or dedicated 5hmC-sensitive methods; assay for transposase-accessible chromatin using sequencing (ATAC-seq); chromatin immunoprecipitation sequencing (ChIP-seq); cleavage under targets and release using nuclease (CUT&RUN); cleavage under targets and tagmentation (CUT&Tag); chromosome conformation capture approaches such as Hi-C; RNA sequencing (RNA-seq); small-RNA sequencing; and, increasingly, single-cell or multimodal profiling (19–21). Functionally, they influence whether genes are poised, repressed, inducible or persistently active in a given cell state. In RA, these layers shape the transcriptional potential of synovial fibroblasts, macrophages, T cells, and B cells. FLS acquire programs linked to migration, invasion, matrix degradation, cytokine production, and apoptosis resistance; myeloid cells adopt inflammatory and tissue-remodeling states; T cells alter activation and differentiation programs; and B cells contribute antigen presentation, autoantibody production, and lymphoid organization (9, 11, 22, 23).

At single-cell resolution, transcriptomic, chromatin-accessibility, and multimodal profiling resolve these cell-specific programs into discrete regulatory states, including inflammatory and tissue-damaging fibroblast populations, activated myeloid programs, lymphoid-organizing synovial niches, and flare-associated circulating precursor-like populations (9–11, 24–26). Joint-selective stromal behavior may also reflect developmental programs and location-specific signaling, including HOX-associated identity and JAK-STAT tuning. HOX genes encode conserved developmental transcription factors that establish positional identity during embryogenesis and can retain differential expression in adult mesenchymal cells. In RA fibroblast-like synoviocytes, differences in DNA methylation, chromatin accessibility, histone marks, and transcriptomic profiles have been reported at HOX loci, including HOXD10, suggesting persistence of an epigenetic memory of anatomical origin.

In parallel, JAK-STAT signaling converts cytokine cues, especially IL-6-family signals, into transcriptional programs through JAK kinases and STAT transcription factors. Hip- and knee-derived RA FLS differ in IL-6/JAK-STAT pathway methylation and functional responsiveness, including IL-6-induced CCL2/MCP1 expression, STAT3 phosphorylation, and JAK1 expression (27–29). These findings motivate a comprehensive synthesis of epigenetic mechanisms in RA and their translational implications. The present review is intended for both readers who know RA but require orientation in epigenetic mechanisms and readers familiar with epigenetics who require RA-specific clinical and immunological context. To avoid treating these mechanisms as parallel catalogs, this review uses the pathogenic regulatory state as its integrating framework. We define a pathogenic regulatory state as a cell- and context-specific configuration of DNA methylation and hydroxymethylation, chromatin accessibility, histone modifications, three-dimensional genome organization, ncRNA circuitry, and RNA modification that determines the transcriptional competence, persistence, and functional behavior of an RA-relevant cell population.

**Figure 1:**
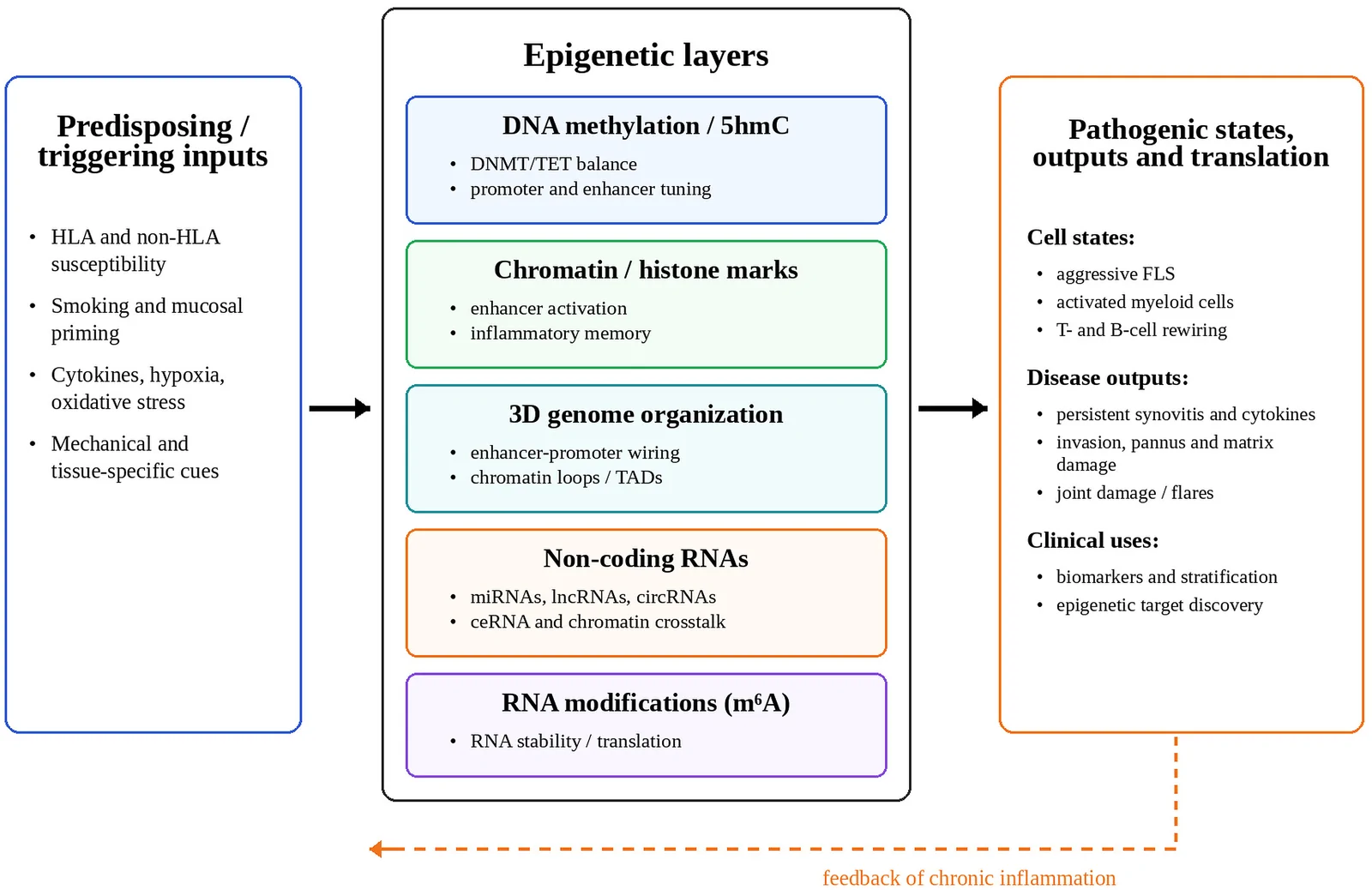
Schematic overview of multilayer epigenetic regulation in rheumatoid arthritis. RA, rheumatoid arthritis; HLA, human leukocyte antigen; 5hmC, 5-hydroxymethylcytosine; DNMT, DNA methyltransferase; TET, ten-eleven translocation enzymes; TAD, topologically associating domain; m6A, N6-methyladenosine; miRNA, microRNA; lncRNA, long non-coding RNA; circRNA, circular RNA; ceRNA, competing endogenous RNA; FLS, fibroblast-like synoviocytes. The figure illustrates how genetic susceptibility and environmental or inflammatory exposures converge on DNA methylation and hydroxymethylation, histone modifications and chromatin accessibility, three-dimensional genome organization - including enhancer-promoter wiring through chromatin loops and TADs - non-coding RNAs, and N6-methyladenosine RNA modification. These interconnected layers establish cell-state- and joint-specific programs in fibroblast-like synoviocytes and immune cells that promote persistent synovitis, tissue invasion, and disease heterogeneity. The translational outputs include biomarker development, patient stratification, flare monitoring, and therapeutic targeting of epigenetic or RNA-regulatory pathways.

The molecular layers and their positions within this regulatory-state framework are summarized schematically in **Figure 1**.

## 2 Major epigenetic layers shaping pathogenic regulatory states in rheumatoid arthritis

### 2.1 DNA methylation and hydroxymethylation: establishing and remodeling regulatory states

Within the regulatory-state framework, DNA methylation and hydroxymethylation provide a comparatively durable layer that can stabilize cellular identity while remaining responsive to genetic and inflammatory inputs. DNA methylation at cytosine residues in CpG dinucleotides is among the best-characterized epigenetic marks. It is often associated with transcriptional repression when located in promoters, but its effect is more context dependent in gene bodies, enhancers, and distal regulatory elements. DNA methylation patterns are established and maintained by DNA methyltransferases (DNMTs) and can be actively remodeled through ten-eleven translocation (TET)-mediated oxidation and downstream repair pathways, generating intermediates such as 5-hydroxymethylcytosine. In immune-mediated disease, including systemic lupus erythematosus, psoriasis, inflammatory bowel disease, multiple sclerosis, and allergic airway inflammation, persistent environmental or inflammatory exposure can leave durable methylation and chromatin imprints that outlast the original stimulus (14, 30).

Conventional bisulfite-based epigenome-wide assays quantify modified cytosine but do not reliably distinguish 5-methylcytosine (5mC) from 5-hydroxymethylcytosine (5hmC) unless dedicated oxidative, enzymatic, or affinity-based approaches are applied (31–33). This distinction is especially relevant in RA because 5hmC is increasingly viewed not only as a transient intermediate of active demethylation but also as a regulatory mark enriched at transcriptionally permissive genes and enhancers (34–36). Accordingly, some methylation changes interpreted as ‘loss of methylation’ in inflammatory tissue may partly reflect TET-dependent hydroxymethylation in activated cell states rather than simple passive demethylation (30, 36). The study by Liu et al. provides a clear example of how RA methylation can be linked to genetic risk rather than treated only as a secondary consequence of inflammation (12). In an epigenome-wide association study (EWAS) of anti-citrullinated protein antibody (ACPA)-positive RA, the authors profiled DNA methylation using Illumina HumanMethylation450 arrays in 354 ACPA-positive RA cases and 337 controls, adjusted for blood-cell heterogeneity and integrated genotype with methylation data. The main signal localized to two clusters in the major histocompatibility complex (MHC) region. Mediation analyses supported a model in which genetic variants influence local methylation states that in turn associate with RA susceptibility, and selected CpGs were replicated in monocyte fractions from an independent cohort. This design does not prove causality, but it provides stronger evidence than a disease-only EWAS because genotype, methylation, and disease status were analyzed together (12).

Purified-cell studies expanded this blood-based view to defined immune subsets and early clinical states. Glossop et al. identified approximately 2,000 differentially methylated CpGs in each of T and B lymphocytes from treatment-naive early RA. Smaller methylation signatures in each cell type separated early RA patients from controls and partially overlapped with established disease (37). Pitaksalee et al. focused on early, drug-naive RA and profiled naive CD4+ T cells, memory CD4+ T cells, and monocytes. Differentially methylated CpGs mapped to 1,047 genes in naive CD4+ T cells, 913 genes in memory CD4+ T cells, and 177 genes in monocytes. Naive CD4+ T cells showed promoter-associated changes in more than 500 genes, including cytokine-related pathways, TNF-region hypomethylation and altered expression of IL21, IL34, and RANKL (38). de la Calle-Fabregat et al. in early arthritis linked blood and synovial monocyte methylomes to prognosis, disease evolution, and treatment-related change (39). These studies also illustrate why analytical strategy is critical. Whole-blood methylation signals can reflect true disease-associated methylation within a cell type, but they can also reflect different proportions of neutrophils, lymphocytes, monocytes, and other leukocyte subsets. Computational deconvolution estimates cellular composition in bulk data: purified-cell designs directly profile sorted T cells, B cells, monocytes, or FLS, and single-cell or multimodal approaches assign regulatory states to individual populations. These strategies do not remove all confounding, but they reduce the risk of mistaking altered cell abundance for altered methylation within a cell type (37, 40–42). Cross-study concordance is incomplete, and proposed diagnostic classifiers remain discovery-stage until performance is reproduced across ancestry, disease stage, treatment exposure, and analytical platforms (43).

Within the joint, synovial fibroblasts display distinctive methylation profiles that correlate with their aggressive, invasive phenotype. In this context, an invasive phenotype refers to the ability of RA FLS to proliferate, resist apoptosis, migrate, secrete cytokines and chemokines, produce matrix-degrading enzymes, and invade cartilage or extracellular matrix (8, 22, 23). Comparative methylome studies provide specific examples. Nakano et al. reported that RA FLS segregate from osteoarthritis and normal FLS on the basis of DNA methylation and identified hypomethylated loci in RA-relevant genes including CHI3L1, CASP1, STAT3, MAP3K5, MEFV, and WISP3. Hypomethylated genes were enriched in pathways related to cell adhesion, extracellular matrix interaction, immune signaling, and cell migration, linking the methylome signature to mechanisms that plausibly support pannus formation and tissue invasion (44). Ai et al. later integrated methylation, histone marks, chromatin accessibility, and transcriptomes in RA FLS and identified disease-associated regulatory regions enriched in inflammatory, matrix-remodeling, and migratory programs, including enhancer/promoter-associated regions marked by active chromatin features (27, 45). Joint location adds another layer of specificity. Ai et al. showed that DNA methylation and transcriptome signatures not only distinguish RA FLS from osteoarthritis FLS but also distinguish RA FLS isolated from knees and hips. Knee-hip differences involved pathways such as IL-6/JAK-STAT signaling, and double-evidenced genes that were both differentially methylated and differentially expressed included multiple HOX genes (27). Ge et al. extended this idea by showing that RA risk variants can have joint-specific effects on target gene expression in FLS, while Lee et al. further emphasized joint-specific regulation of HOXD10. Together, these data support the idea that anatomical identity, local cytokine exposure, and chromatin architecture can influence which programs become pathogenic in a given joint (13, 29).

Mechanistically, several routes may contribute to the hypomethylated RA FLS state. Increased recycling of polyamines in RA synoviocytes has been linked to depletion of S-adenosylmethionine, thereby limiting methyl-group availability for DNMT-dependent reactions and promoting widespread hypomethylation (46). Proinflammatory cytokines can also repress the methylation machinery: IL-1β rapidly decreases DNMT1 and DNMT3A expression in synoviocytes. In prolonged IL-1β stimulation models, this exposure induces a subset of methylation and transcriptional changes that overlap with RA FLS signatures, suggesting that chronic cytokine exposure can contribute to, but is unlikely to fully explain, stable stromal epigenetic remodeling (44, 47). Methylation remodeling also appears to begin very early in disease evolution. Synovial fibroblasts obtained before definitive RA classification already show disease-associated methylation changes at loci related to adhesion, matrix interaction, and immune signaling. This suggests that stromal epigenetic reprogramming may precede, or at least accompany, the transition from at-risk arthralgia and early inflammatory arthritis to persistent RA (48).

Early methylation remodeling helps explain why RA fibroblasts retain an aggressive, quasi-transformed phenotype *in vitro* after removal from the joint environment and supports the broader concept of inflammatory memory in tissue stromal cells (8, 9, 49). In this review, inflammatory memory refers to a persistent, epigenetically encoded state in which prior inflammatory stimulation leaves synovial stromal cells poised for sustained or exaggerated inflammatory, migratory, and tissue-destructive responses after the initiating stimulus has subsided (49–51). This concept overlaps mechanistically with, but is not synonymous with, trained immunity. Trained immunity describes long-term functional reprogramming of innate immune cells or their progenitors, typically involving coordinated epigenetic and metabolic changes that alter responses to a later, often unrelated, challenge (52). By contrast, the term inflammatory memory is used here more broadly for durable stimulus-induced changes in tissue-resident stromal cells, particularly RA FLS, and does not imply antigen-specific adaptive memory. This distinction is descriptive rather than categorical (49). Evidence for reproducibility is mixed even among the best-characterized RA methylation studies. Liu et al. identified two MHC-associated methylation clusters in 354 ACPA-positive RA cases and 337 controls; however, in an independent CD14+ monocyte cohort of 12 cases and 12 controls, each cluster was supported by at least one CpG at P < 0.01, whereas the remaining candidate CpGs showed only suggestive associations. As noted above, only a subset of the early-RA T- and B-cell methylation changes showed comparable alterations in established disease. These findings support the existence of reproducible cell- and stage-specific methylation programs, while cautioning against treating individual CpGs as universal RA markers across cellular compartments and disease stages (12, 37). Programs reported in circulating immune cells, monocytes, and synovial fibroblasts overlap only partly, and recent whole-blood EWAS replicated only a subset of previously reported loci (53). This partial reproducibility is encouraging but also demonstrates that discovery signals are sensitive to smoking, age, sex, serostatus, leukocyte composition, treatment exposure, ancestry, platform, and disease stage (40–43, 53). A locus-level association should therefore be considered robust only after independent replication in a comparable cell type and clinical context.

Hydroxymethylation adds a functionally distinct layer. In one integrated human and mouse study, TET3 dioxygenase expression and global 5hmC abundance were increased in RA synovium and RA FLS, and TNF-α can further enhance both, directly linking inflammatory signaling to active DNA demethylation dynamics (36). In human RA FLS, increased expression of the TET3 dioxygenase promotes migration and upregulates inflammatory mediators such as CXCL8 and CCL2. Partial Tet3 deficiency attenuated arthritis severity in the study’s mouse model, supporting a functional contribution rather than a purely descriptive association (36).

At the translational level, the central question is whether a methylation or hydroxymethylation feature identifies a stable pathogenic regulatory state, a reversible treatment-responsive state, or a secondary inflammatory scar. Rapidly shifting marks may serve as pharmacodynamic indicators, whereas reproducible stable signatures may better capture predisposition, chronic inflammatory imprinting, or relapse risk. Prospective studies should combine serial blood and synovial sampling, sorted or single-cell populations, imaging, and 5mC/5hmC-resolving assays to test prespecified associations with flare, non-response, or erosive progression (24, 39, 43, 53). Across the current literature, evidence is strongest for cell-type- and context-specific programs rather than a universal RA methylome. Cohorts remain modest, ancestry diversity is incomplete, most associations are non-causal, and bulk platforms can obscure cell-state heterogeneity. Nonreplicated loci and null associations should be reported explicitly because they help distinguish robust state markers from study-specific signals. **Table 1** summarizes representative findings together with their principal interpretive limitations.

**Table 1:** Representative DNA methylation and hydroxymethylation alterations in rheumatoid arthritis, with study-level citations and principal interpretive limitations.

Dimension	Representative RA-associated finding and study-level citations	Main compartment	Translational relevance and principal limitation/replication status
Blood methylation signatures	Cell-composition-aware EWAS identify differential methylation in whole blood, including recent-onset and ACPA-positive disease, and support emerging classifier models (12, 43, 53, 54).	Whole blood/PBMCs	Accessible and scalable, but strongly affected by leukocyte composition, smoking, treatment, ancestry, and disease stage. Locus-level concordance is partial, and classifier performance requires external validation across populations and platforms.
Synovial fibroblast hypomethylation	RA fibroblast-like synoviocytes show broad methylome remodeling and relative global hypomethylation compared with osteoarthritis or non-inflamed controls (27, 44, 45)	Synovium/FLS	Supports aggressive, cytokine-producing, apoptosis-resistant fibroblast states and inflammatory memory. Pathway-level convergence is stronger than replication of individual loci across cohorts or joint sites.
Joint-specific epigenetic imprinting	Methylation and transcriptomic programs differ by anatomical site, indicating that local tissue context shapes synovial fibroblast identity (27–29).	FLS from distinct joints	May contribute to clinical heterogeneity and location-dependent responses. Evidence is strongest for hip-knee comparisons; replication across other joints and independent cohorts is limited.
Early disease remodeling	Differential methylation can be detected in treatment-naive early RA and may already emerge before fully established chronic synovitis (37–39, 48).	Early RA/at-risk states	Supports involvement in disease evolution rather than only a secondary inflammatory scar. Cohorts are generally modest, and signals remain cell-type- and disease-stage-dependent.
Loss of methylation control	Altered methyl-donor metabolism and cytokine-associated reduction of DNMT expression/activity have been linked to impaired maintenance methylation in RA synoviocytes (46, 47).	RA FLS	Provides a mechanistic explanation for stable hypomethylation. Evidence is mainly ex vivo or stimulation-based, with limited independent *in vivo* patient-level replication.
TET3/5hmC axis	TET3 expression and global 5hmC are increased in RA synovium and RA FLS, and TNF-α can further amplify this program (36).	RA synovium/FLS	Functionally links hydroxymethylation to chemokine production, migration, and arthritis severity. Independent replication in additional human cohorts and cell-type-resolved 5hmC datasets is limited.

RA, rheumatoid arthritis; ACPA, anti-citrullinated protein antibodies; EWAS, epigenome-wide association study; FLS, fibroblast-like synoviocytes; PBMCs, peripheral blood mononuclear cells; 5hmC, 5-hydroxymethylcytosine.

### 2.2 Chromatin accessibility and histone modifications: activation and persistence of regulatory states

Within a pathogenic regulatory state, histone modifications and chromatin accessibility form the comparatively dynamic layer that determines which loci are transcriptionally competent and how rapidly they respond. Acetylation and methylation of histone tails (for example, H3K27ac at active enhancers and promoters, H3K4me1 at enhancers, and H3K4me3 at promoters) correlate with transcriptional competence by modulating nucleosome stability and by recruiting reader proteins. These marks do not act in isolation; rather, they form combinatorial promoter and enhancer states that can be measured by ChIP-seq, CUT&RUN, CUT&Tag, and ATAC-seq and integrated with RNA-seq to infer active regulatory programs (16–18, 30). Comprehensive epigenomic profiling of synovial fibroblasts has revealed that RA FLS harbor extensive alterations in enhancer landscapes compared with controls. Multilayer mapping of histone marks, chromatin accessibility, and transcriptomes identified differentially modified enhancer regions that cluster in pathways central to inflammation, matrix remodeling, and cell migration (45). Systems-level analyses further supported the existence of biologically distinct FLS states and regulatory networks, suggesting that heterogeneity in transcription factor (TF) activity contributes to variable inflammatory outputs (55).

Inflammatory memory represents persistence of this chromatin-enabled state beyond the initiating stimulus. TNF exposure induces chromatin activation at regulatory elements of inflammatory genes that escape repression, accompanied by sustained enhancer acetylation and prolonged gene expression kinetics in RA FLS (49). Inflammatory cytokines can also remodel the expression of chromatin regulators themselves. For example, suppression of HDAC5 by IL-1β or TNF promotes type I interferon response gene transcription in RA FLS and links extracellular inflammation to intracellular chromatin-regulatory balance (51). Mechanistically, enhancer acetylation is written by histone acetyltransferases and interpreted by bromodomain and extra-terminal (BET) proteins, which can assemble transcriptional machinery at acetylated chromatin. Functional studies have implicated CBP/p300 and BET proteins in the regulation of inflammatory gene programs in synovial fibroblasts. Pharmacological BET inhibition can also dampen cytokine-induced responses, supporting the concept that chromatin readers represent therapeutic entry points (56–59).

However, because chromatin regulators are broadly expressed and participate in multiple physiological pathways, specificity, dosing, and tissue targeting remain key translational barriers (60). Recent single-cell and multimodal approaches provide the needed resolution by linking chromatin accessibility to discrete synovial cell states. Multi-omic studies have identified activated FLS programs linked to specific transcription-factor motifs and pathogenic myeloid programs associated with tissue inflammation and damage (10, 11, 25, 26).

Together, these datasets support a state-based interpretation of RA heterogeneity, but they do not establish that every accessible or acetylated region is causal. Most studies profile selected synovial-fibroblast cohorts or experimental stimulation models, and cross-cohort replication of individual regulatory elements remains limited. Pharmacological suppression of cytokine-responsive chromatin can support mechanism, yet it may also reflect broad interference with homeostatic transcription. The most credible regulatory-state assignments will therefore require convergence among accessibility, histone marks, expression, perturbation, and reproducibility in independent cell-type-resolved cohorts.

### 2.3 Three-dimensional genome organization: linking RA risk to state-specific effector genes

Three-dimensional genome organization supplies the spatial wiring of a pathogenic regulatory state. Chromatin loops and topologically associating domains (TADs) constrain enhancer/promoter communication and enable distal regulatory elements to act on target genes over long genomic distances (61, 62). In RA, this is especially relevant because many risk loci lie in non-coding regulatory DNA and therefore require 3D mapping to identify their effector genes. Methods such as Hi-C, capture Hi-C, and promoter-capture approaches complement ATAC-seq and ChIP-seq by connecting disease-associated enhancers to promoters in the relevant cell type. Hi-C surveys genome-wide contacts but often at modest resolution, whereas capture Hi-C and promoter-capture Hi-C enrich selected regulatory fragments or promoters to improve detection of enhancer-promoter links. This distinction matters for RA because assigning a non-coding variant to the nearest gene can be misleading when the physical promoter contact points to a more distant immune or stromal effector gene (13, 62, 63).

Ge et al. integrated DNA architecture, 3D chromatin interactions, chromatin accessibility, and gene expression in synovial fibroblasts, B cells, and T cells with genetic fine mapping of RA loci. This work mapped putative causal variants, enhancers, genes, and cell types for approximately 30-60% of RA loci and estimated that fibroblast-like synoviocytes (FLS) may account for up to 24% of RA heritability. Tumor necrosis factor (TNF) stimulation also altered topologically associating domains, chromatin state and expression of putative causal genes such as TNFAIP3 and IFNAR1, demonstrating how inherited risk and inflammatory stimulation can converge within the synovial stromal compartment (13).

Even with 3D mapping, causal assignment should remain cautious. A chromatin loop shows that two regulatory regions can interact, but it does not establish functional regulation. Candidate enhancer-gene assignments may differ across cell types, joint sites, stimulation conditions, and analytical platforms, and many reported contacts have not been independently perturbed or replicated. Stronger inference requires allele-specific effects, enhancer perturbation, expression changes in the relevant cell state, and concordance with methylation or chromatin-accessibility data. Functional genomics can prioritize candidate genes and pathways, but it cannot by itself prove that a locus is causal in every patient or joint.

Joint-specific epigenomic regulation adds further complexity. Location-dependent enhancer usage, HOX gene regulation, and chromatin interaction indicate that the same genetic variant or cytokine signal can have different consequences depending on anatomical context and FLS identity (27, 29). Within the regulatory-state framework, 3D genome maps are therefore best viewed as hypotheses about context-specific wiring. Their translational value lies in prioritizing genes and pathways for perturbation, not in treating physical proximity as sufficient evidence of causality. Robust inference will require integration of chromatin folding, histone modification, DNA methylation, ncRNA control, and perturbation experiments across immune and stromal cell states (13).

### 2.4 Non-coding RNAs: state regulators, cross-layer connectors, and biomarkers

Within the regulatory-state framework, ncRNAs are considered by function rather than as an inventory of molecules: intracellular effectors that tune signaling, cross-layer connectors that interact with DNA and histone regulation, and extracellular readouts that may report tissue states. The examples below are selected to illustrate these roles and the strength of evidence supporting them. Non-coding RNAs (ncRNAs) are RNA molecules that are not translated into proteins but can regulate gene expression at transcriptional, post-transcriptional, and chromatin levels. Major classes discussed in RA include microRNAs (miRNAs), long non-coding RNAs (lncRNAs), circular RNAs (circRNAs), small nucleolar RNAs (snoRNAs), and extracellular or vesicle-associated RNAs. Depending on their structure and localization, ncRNAs can guide or scaffold chromatin-modifying complexes, modulate transcription-factor activity, alter mRNA stability or translation, and act as molecular decoys or sponges. Because their expression is also influenced by DNA methylation and chromatin accessibility, ncRNAs participate in reciprocal regulatory networks that can stabilize pathogenic cell states (64–66).

First, intracellular microRNAs repress target mRNAs post-transcriptionally and thereby tune signaling networks rather than acting as isolated single-gene switches. In RA, deregulated miRNAs converge on pathways that include nuclear factor kappa B (NF-κB), JAK-STAT, mitogen-activated protein kinase (MAPK), apoptosis resistance, cytoskeletal remodeling, and osteoclast-promoting signals (67). A well-studied example is miR-203, which is overexpressed in RA synovial fibroblasts and promotes inflammatory mediator production, illustrating how miRNA dysregulation can contribute to the stable aggressive phenotype of RA FLS (68). Likewise, miR-146 has been detected in RA synovial tissue and is implicated in feedback regulation of innate immune signaling, although its net effect varies with cellular and network context (69). Methotrexate also alters miRNA expression in RA FLS, supporting the possibility that miRNA panels may serve as pharmacodynamic biomarkers (70). A mechanistically informative example is the microRNA-22 (miR-22; MIR22)-TET3-MTRNR2L2 axis. In RA FLS, MIR22 directly represses TET3 translation by binding to the 3′ untranslated region of TET3, whereas TET3 promotes 5-hydroxymethylcytosine (5hmC) at the MTRNR2L2 promoter and supports a proinflammatory fibroblast state (71). Thus, MIR22 acts as an inhibitory upstream regulator of the proinflammatory TET3-MTRNR2L2 arm. This axis directly links a miRNA to DNA hydroxymethylation and illustrates the multilayer nature of RA epigenetic regulation. The evidence hierarchy should nevertheless be considered carefully: expression differences establish association, gain- or loss-of-function experiments support mechanism, and diagnostic or prognostic panels require independent validation in appropriately controlled cohorts (72, 73). Thus, miR-203 and the MIR22-TET3-MTRNR2L2 axis have functional FLS-centered evidence, whereas many circulating miRNA studies primarily show associations with RA status, activity, or treatment response (68, 71, 74, 75).

Second, chromatin-linked lncRNAs illustrate how RNA can encode anatomical context and modify regulatory machinery. HOTAIR is expressed predominantly in knee synovial fibroblasts and contributes to more than half of the joint-specific transcriptional program in these cells. It is downregulated by proinflammatory cytokines and is expressed at lower levels in RA knee samples than in osteoarthritis. Functional studies indicate that HOTAIR modulates phosphoinositide 3-kinase (PI3K)-Akt, IL-6, and Wnt-related pathways, together with processes linked to migration, osteoclastogenesis, and immune-cell recruitment. These findings suggest that lncRNAs can encode positional identity within the synovium and may help explain why RA pathology is not uniform across anatomical sites (76). Other lncRNAs provide direct examples of crosstalk with chromatin-regulatory enzymes. NEAT1 knockdown alleviates inflammatory features of RA in part through repression of the p300/CBP axis and reduced IL-18 expression, linking an lncRNA to histone-acetylation-associated transcriptional machinery (77). PVT1 has been linked to DNA methylation control through regulation of SIRT6 methylation status, and its silencing suppresses inflammatory activity while promoting apoptosis in RA FLS (78).

Competing endogenous RNA (ceRNA) regulation provides another mechanism through which lncRNAs can influence the aggressive RA FLS phenotype. In a ceRNA network, an RNA molecule binds, or ‘sponges,’ a shared miRNA and thereby reduces miRNA-mediated repression of other transcripts. GAPLINC is upregulated in RA FLS and promotes proliferation, migration, invasion, and inflammatory responsiveness through such a miRNA-sponge mechanism (79). However, ceRNA models can be overinterpreted because biologically meaningful competition requires the participating RNAs to be sufficiently abundant and present in compatible subcellular compartments. Under these conditions, selected lncRNA–miRNA–mRNA modules may influence fibroblast survival and pannus-like behavior (76–79).

Third, snoRNAs and other non-canonical ncRNAs further broaden this regulatory landscape. In a mouse model *Snord3* promoted arthritis through epigenetic regulation of ESM1 in FLS and identified enhancer of zeste homolog 2 (EZH2) as the chromatin-regulatory intermediate in an SNORD3-EZH2-ESM1 axis (80). ESM1 denotes endothelial cell-specific molecule 1, also known as endocan, a secreted proteoglycan originally characterized as an endothelial marker; in the cited study, it acted as a downstream effector in FLS rather than merely as a marker of endothelial cells.

Fourth, extracellular and vesicle-associated ncRNAs are attractive from a biomarker perspective because they may report intercellular communication as well as disease state (81). Plasma exosomal miR-204-5p is reduced in RA and correlates inversely with serological and inflammatory indices such as rheumatoid factor (RF), erythrocyte sedimentation rate (ESR), and C-reactive protein (CRP). It can also be transferred functionally from T cells to synovial fibroblasts, where it restrains proliferation and inflammatory activation (82). Experimentally, restoring miR-204-5p ameliorates arthritis severity, supporting both mechanistic and biomarker relevance (82). Likewise, circulating miR-19b has been linked to disease progression and to response to baricitinib, illustrating that blood-based ncRNA signatures may capture dynamic treatment effects rather than merely static diagnosis (70, 83).

CircRNAs are emerging as a complementary biomarker class because their covalently closed structure confers high stability in plasma and extracellular vesicles (84). Exosomal hsa_circ_0003914 has been proposed as a novel RA biomarker and RA-derived plasma exosomes enriched for this signal aggravated collagen-induced arthritis *in vivo*, suggesting that some circulating circRNAs may be functionally active rather than passive byproducts (75). A similar limitation applies to recently proposed circRNA signatures. Yang et al. reported diagnostic AUCs ranging from 0.794 to 0.993 for six circRNAs; however, the discovery analysis included only three treatment-naïve patients with RA and three healthy controls, followed by RT-qPCR evaluation in a separate within-study cohort of 32 patients and 32 controls. Because the candidates have not yet been tested in an external multicenter cohort, these findings should be regarded as promising within-study validation rather than independent replication (85). Even so, circRNAs may become valuable components of multimarker panels because their stability is advantageous for real-world sample handling.

The examples above do not imply uniform support across ncRNA classes. The literature remains concentrated on a small set of repeatedly studied molecules, whereas broader sequencing suggests additional PIWI-interacting RNAs, tRNA-derived fragments, Y-RNAs, and small nuclear RNA derivatives.

Some of these classes may prove more stable in blood or more informative of cellular origin than canonical miRNAs (86). This imbalance creates a risk of citation and publication bias: highly positive candidates are repeatedly discussed, whereas null or discordant results are less visible (87). Concrete discordant and null findings have also been reported in circulating-miRNA studies. Murata et al., in a cohort of 102 patients with RA and 104 healthy controls, reported increased plasma miR-26a in RA and obtained a sensitivity of 53.9% and specificity of 94.3% at the selected diagnostic cutoff. In contrast, Cieśla et al., studying 50 patients with RA and 24 controls, found lower plasma miR-26a concentrations in RA than in healthy controls. In the latter study, miR-93 did not differ significantly between patients and controls, and none of the measured miRNAs was independently associated with DAS28 in the multivariable model. These opposing and null findings illustrate how disease stage, treatment exposure, cohort composition, normalization strategy, and preanalytical procedures may alter both the direction and apparent clinical performance of an ncRNA signal (88, 89).

From a therapeutic standpoint, ncRNAs are attractive because they may sit upstream of several inflammatory outputs, but the same network centrality raises specificity and safety concerns (90). Priority should therefore be given to candidates with reproducible directionality, clearly defined cellular origin, and experimentally validated links to pathogenic pathways (71, 76, 77, 82). Even for these candidates, clinical utility remains unproven.

Across these categories, clinical translation of ncRNA biomarkers still faces substantial obstacles. Results vary according to biospecimen type (whole blood, peripheral blood mononuclear cells (PBMCs), serum, plasma, synovial fluid, or purified exosomes), RNA-isolation protocols, normalization strategy, hemolysis control, and treatment status. For extracellular RNA studies, preanalytical control is particularly important because platelet activation, hemolysis, delayed processing, freeze-thaw cycles, and extracellular-vesicle isolation methods can all change the apparent RNA profile. Reporting standards for extracellular vesicle studies are therefore relevant to RA biomarker work, even when the primary question is immunological rather than vesicle biology (91). In addition, many proposed markers have been evaluated only in modest single-center cohorts (74, 75, 82, 83). Consequently, a positive association or high discovery AUC should not be treated as evidence of disease specificity or clinical readiness. Discordant or null replication may reflect technical variation, biological heterogeneity, or an initially optimistic estimate, and each possibility should be reported rather than obscured by pathway-level synthesis. Composite panels may ultimately be more realistic than a single universal marker, but they must be evaluated against clinically relevant comparators and simpler clinical models (72, 73).

Circulating ncRNAs are analytically attractive because ribonucleoprotein complexes and membrane vesicles can protect them from degradation, enabling repeated and retrospective sampling. Biological source attribution nevertheless remains difficult: a plasma RNA signal may arise from activated immune cells, endothelium, platelets, or synovial tissue, and the relative contribution of these compartments may shift with therapy, infection, or flare (64, 82, 83). For this reason, the field is increasingly moving toward paired profiling of cell-associated and extracellular ncRNAs, ideally linked to single-cell atlases of synovium and blood. Such designs may clarify whether a candidate circulating RNA is simply correlated with inflammation or instead serves as a faithful surrogate of a specific pathogenic tissue program. Coordinated patterns across RNA classes may encode such states more precisely than a single transcript (75, 82, 83). The translational goal is therefore not simply to maximize discrimination in a discovery set, but to demonstrate that an accessible RNA signature retains directionality, calibration, and added clinical value under prespecified external validation. Mechanistically anchored combinations—for example, pairing a circulating RNA with a methylation feature—may improve interpretability, but they remain hypotheses until tested prospectively.

**Figure 2:**
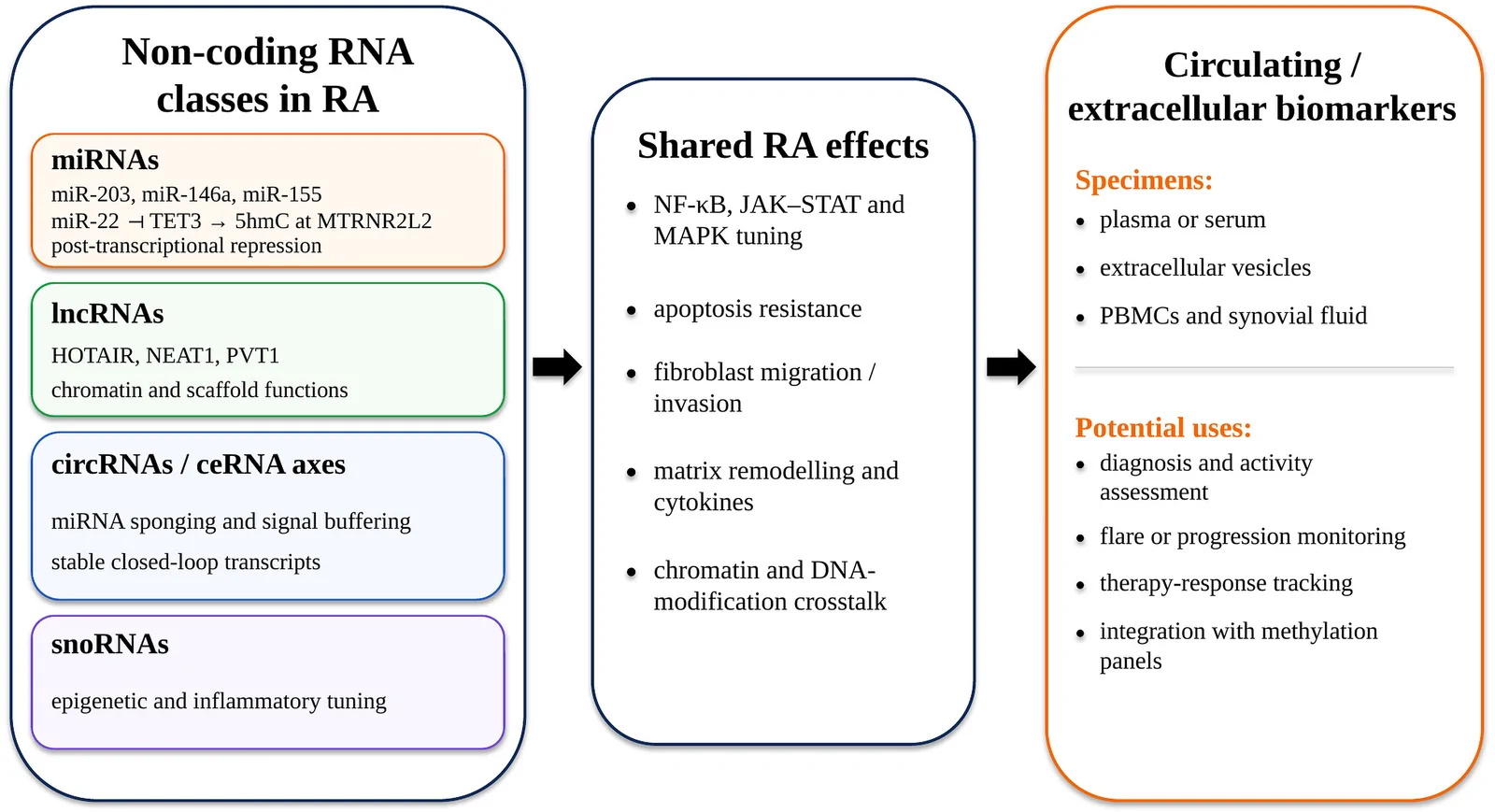
Non-coding RNAs as intracellular effectors and extracellular biomarkers in rheumatoid arthritis. RA, rheumatoid arthritis; ncRNA, non-coding RNA; miRNA, microRNA; lncRNA, long non-coding RNA; circRNA, circular RNA; ceRNA, competing endogenous RNA; snoRNA, small nucleolar RNA; NF-κB, nuclear factor kappa-light-chain-enhancer of activated B cells; JAK-STAT, Janus kinase-signal transducer and activator of transcription; MAPK, mitogen-activated protein kinase; PBMCs, peripheral blood mononuclear cells; HOTAIR, HOX transcript antisense intergenic RNA; NEAT1, nuclear paraspeckle assembly transcript 1; miR-22, microRNA-22; 5hmC, 5-hydroxymethylcytosine; MTRNR2L2, MT-RNR2-like 2; TET3, ten-eleven translocation 3. The figure summarizes the principal ncRNA classes discussed in Section 2.4 and separates intracellular mechanisms from extracellular biomarker applications. Intracellular miRNAs, lncRNAs, circRNAs, and snoRNAs modulate mRNA stability and translation, ceRNA interactions, chromatin-modifying enzymes, and inflammatory signaling pathways, with representative examples including HOTAIR, NEAT1, the miR-22-TET3 axis, and Snord3-ESM1 signaling in fibroblast-like synoviocytes. Extracellular or vesicle-associated ncRNAs detected in blood-derived specimens are shown as candidate markers of diagnosis, disease activity, progression, and treatment response. In the miR-22–TET3–MTRNR2L2 axis, the inhibitory symbol (⊣) denotes repression, whereas the arrow (→) denotes a positive downstream regulatory effect. Accordingly, miR-22 ⊣ TET3 indicates that miR-22 represses TET3 translation, while TET3 → 5hmC at MTRNR2L2 indicates that TET3 promotes 5-hydroxymethylcytosine at the MTRNR2L2 promoter and supports a proinflammatory FLS program. Other arrows denote proposed regulatory or intercellular communication relationships and should not be interpreted as proof of causality in every patient.

Key intracellular ncRNA effectors and circulating biomarker candidates highlighted in Section 2.4 are synthesized in **Figure 2** and **Table 2**.

**Table 2:** Selected non-coding RNAs and ncRNA classes implicated in rheumatoid arthritis pathogenesis and biomarker development, with study-level citations and replication status.

ncRNA or class	Representative RA-linked mechanism and study-level citations	Main compartment	Relevance and principal limitation/replication status
miR-203	Overexpression in RA synovial fibroblasts promotes inflammatory mediator production and supports an aggressive stromal phenotype (68).	Synovium/FLS	Mechanistic FLS evidence is established, but this is not an externally validated clinical biomarker; independent patient-level biomarker replication remains limited.
Inflammatory miRNAs (e.g., miR-146, miR-155)	Altered miRNA programs tune innate and cytokine signaling in synovial and immune cells, and methotrexate changes miRNA expression in RA FLS (69, 70).	Synovium/PBMCs	Effects are cell- and network-context-dependent. Their most realistic use is within panels rather than as universal single-analyte markers, and cross-cohort replication is uneven.
HOTAIR and other context-dependent lncRNAs	HOTAIR contributes to joint-specific transcriptional programs and links positional identity to inflammatory and migratory behavior in synovial fibroblasts (76).	FLS/joint-specific synovium	Highlights anatomical specificity of lncRNA effects. Evidence is compelling in the studied joint contexts but requires replication across additional joints, cohorts, and disease stages.
NEAT1	NEAT1 knockdown reduces inflammatory features in RA models partly through repression of the p300/CBP axis and IL-18 (77).	RA FLS/experimental models	Candidate upstream regulator, but evidence remains predominantly experimental; independent clinical validation and assessment of cell-type specificity are limited.
miR-22/MIR22–TET3–MTRNR2L2 axis	miR-22 directly represses TET3 translation by binding to the TET3 3′ untranslated region. TET3 promotes 5-hydroxymethylcytosine at the MTRNR2L2 promoter and supports a proinflammatory FLS state (71).	Synovium/FLS; arthritis models	Supported by RA-FLS experiments and mouse arthritis models in one primary study; independent clinical replication is lacking.
circRNAs/ceRNA networks	circRNAs may act through miRNA-sponging/ceRNA mechanisms and are being evaluated as stable circulating biomarkers; examples include hsa_circ_0003914 and recent diagnostic signatures (75, 85).	Synovium/blood/EVs	High stability is attractive, but several diagnostic studies use very small discovery cohorts and lack external multicenter validation. ceRNA claims also require compatible abundance and localization.
snoRNAs	Snord3 promoted arthritis in a mouse model through an SNORD3-EZH2-ESM1 epigenetic axis in FLS (80).	Experimental FLS/arthritis models	Expands the landscape beyond canonical miRNAs/lncRNAs, but current evidence is preclinical and human replication is limited.
Circulating ncRNA panels	Plasma/serum and extracellular-vesicle ncRNAs have been associated with RA status, activity, progression, or treatment response (75, 82, 83, 88, 89).	Plasma/serum/EVs	Promising for composite panels, but direction can be discordant across studies (e.g., plasma miR-26a); preanalytical variation, treatment effects, small cohorts, and limited external validation remain major constraints.

ceRNA, competing endogenous RNA; circRNA, circular RNA; EVs, extracellular vesicles; FLS, fibroblast-like synoviocytes; lncRNA, long non-coding RNA; miR-22/MIR22, microRNA-22; MTRNR2L2, MT-RNR2-like 2; ncRNA, non-coding RNA; PBMCs, peripheral blood mononuclear cells; TET3, ten-eleven translocation 3.

### 2.5 Epitranscriptomics: dynamic tuning of pathogenic regulatory states

In addition to DNA and histones, chemical modifications of RNA (the epitranscriptome) can influence gene expression by altering mRNA stability, splicing, localization, and translation. N6-methyladenosine (m6A), the most abundant internal mRNA modification, is deposited by methyltransferase (writer) complexes, removed by demethylases (erasers) such as FTO and ALKBH5, and interpreted by reader proteins including YTH-domain factors and IGF2BP family members (92). Emerging RA studies report altered m6A regulators and links to cytokine signaling and synovial activation (93, 94). In parallel, mechanistic work has implicated m6A reader pathways in promoting FLS activation, supporting the idea that posttranscriptional regulation contributes to stable inflammatory programs (94). However, this evidence remains preliminary. The studies mentioned above provide evidence that FTO-mediated m6A regulation can influence synovial aggression and inflammation, or implicate an IGF2BP3-RASGRF1 axis in joint injury. The reported FTO-mediated and IGF2BP3-RASGRF1 axes move beyond descriptive expression, but the number of studies is small, independent replication is limited, and no consistent longitudinal, cell-type-resolved patient signature has been established (93, 94). While the epitranscriptomic layer is still less mature than DNA methylation or chromatin profiling in RA, it offers a conceptually attractive avenue because RNA modifications can, in principle, be rapidly reversible and may provide opportunities for temporally precise interventions. Key priorities include cell-type-resolved mapping of RNA modifications in synovial tissue and integration with chromatin states and genetic risk. It is also important to determine whether m6A changes represent upstream drivers or downstream consequences of inflammatory activation (93).

### 2.6 Translational implications

Translation should focus on reproducible pathogenic regulatory states rather than isolated epigenetic marks. Blood-based methylation signatures and classifier models have been proposed for diagnostic support and risk estimation, and their performance will depend on cohort diversity, disease stage, therapy exposure, cell composition, and comparison with appropriate clinical baselines (43). Clinically, biomarker interpretation depends on the biospecimen. Whole blood and plasma are scalable, but they are vulnerable to shifts in leukocyte subsets, platelet contamination, hemolysis, and treatment exposure. Synovial tissue is mechanistically richer, but it is less accessible and can vary by biopsy site, degree of inflammation, and local tissue composition. These practical differences should be stated when comparing biomarker studies across compartments. Beyond diagnosis, epigenomic data can inform stratification and mechanism-based patient grouping. Single-cell multi-omics has defined synovial regulatory states linked to inflammatory intensity, stromal activation, and tissue organization. These states may serve as molecular endotypes that predict therapeutic response or relapse (10, 11, 24–26). Integrating these state definitions with accessible surrogate markers (for example, circulating DNA methylation features or ncRNA panels) is a critical next step for clinical utility. Therapeutically, chromatin regulators represent candidate targets to reset inflammatory programs and disrupt pathological memory. Pharmacological inhibition of BET proteins, histone deacetylases, and other chromatin enzymes can attenuate inflammatory gene expression in synovial fibroblasts and has shown efficacy in experimental models, motivating translational efforts (56, 57, 59). These findings do not establish clinical benefit, and the pleiotropic roles of chromatin regulators create risks of on-target toxicity and broad immunosuppression. Proposed strategies include targeting reader domains, exploiting state-specific dependencies, and concentrating delivery in inflamed synovium (60).

A complementary use of epigenomic maps is to prioritize conventional drug targets by linking risk variants and disease-associated enhancers to effector genes in specific states (13). This approach is useful for non-coding GWAS loci and may reveal targets in joint or stromal cells that are not found in immune-focused analyses. An example of this logic is the Ge et al. framework, where chromatin contacts and enhancer states link variants to candidate target genes in FLS, B cells, and T cells. Such maps can propose druggable pathways, but target nomination should be followed by perturbation, replication in independent cohorts, and comparison with clinically accessible surrogate biomarkers (13). Crucially, translational studies should predefine the marker, model, threshold, comparator, and failure criteria, and they should report negative or nonreplicated results. Establishing whether a given epigenetic alteration is a driver, a compensatory response, or a readout of a particular state will determine whether it is useful for intervention or monitoring (24, 95–97).

A particularly promising translational direction is the construction of composite epigenetic models that integrate blood-based DNA methylation features with circulating ncRNAs and conventional clinical variables. Single-layer biomarkers rarely capture the full biological diversity of RA, whereas multi-analyte models can combine mechanistic breadth with clinical accessibility. In practice, such algorithms may be especially useful in early or seronegative disease. They may also help monitor subclinical residual activity or identify patients whose apparent clinical similarity masks different underlying regulatory states (43, 54, 74, 83). However, added complexity is justified only when a model improves discrimination, calibration, and clinical decision-making beyond simpler clinical or serological models in an external cohort.

RNA-based interventions, including antisense oligonucleotides, small interfering RNAs, miRNA mimics or inhibitors, and extracellular-vesicle-based delivery platforms, could in principle modulate pathogenic ncRNA circuits upstream of cytokine release and tissue invasion. However, the same issues that complicate biomarker development, including cell-type specificity, biodistribution, off-target effects, and context-dependent network behavior, also apply to intervention (71, 77, 82, 90).

The narrative synthesis also has limitations. It is not a formal systematic review or meta-analysis, and the depth of evidence differs substantially across epigenetic layers. Many studies are small, single-center, cross-sectional, or model-based, while publication and citation bias may make positive findings more visible than null or discordant results (87). Candidate biomarkers may reflect inflammation, treatment exposure or cell redistribution rather than RA-specific causal biology. We therefore regard individual markers as provisional unless they show independent replication in the same biospecimen or cell state, mechanistic coherence, and added value over standard clinical measures. The strength of the review is its multilayer integration of DNA methylation, hydroxymethylation, chromatin state, 3D genome organization, ncRNAs, and RNA modifications; the key translational challenge is to convert this integrative map into validated, cell-type-aware, and clinically actionable biomarkers or targets.

## 3 Conclusions

Epigenetic alterations in RA are most coherently understood as a set of pathogenic regulatory states rather than a catalog of independent marks. Each state is produced by the interaction of DNA methylation and hydroxymethylation, histone modifications, chromatin accessibility, 3D genome organization, ncRNA circuits, and RNA modification. This framework explains how non-coding genetic risk and environmental exposure can yield durable yet potentially reversible programs in synovial fibroblasts and tissue-resident immune cells. Inflammatory memory is one temporal phenotype of such a state, not an alternative organizing principle. The evidence base is nevertheless uneven. Replication is strongest for broad themes: cell-type specificity, FLS remodeling, and context-dependent regulatory wiring, whereas many individual CpGs, ncRNAs, chromatin contacts, and m6A axes remain single-cohort or model-specific. Translation should therefore advance only candidates that show reproducible directionality, mechanistic coherence, and added clinical value in prespecified external studies. On this basis, the near-term opportunity is not a universal epigenetic test or a broadly acting ‘epigenetic drug,’ but state-informed biomarkers and targets that can be linked to defined pathogenic compartments and monitored over time.
